# Statistical analysis plan for a cluster-randomized crossover trial comparing the effectiveness and safety of a flexible family visitation model for delirium prevention in adult intensive care units (the ICU Visits Study)

**DOI:** 10.1186/s13063-018-3006-8

**Published:** 2018-11-19

**Authors:** Daniel Sganzerla, Cassiano Teixeira, Caroline Cabral Robinson, Renata Kochhann, Mariana Martins Siqueira Santos, Rafaela Moraes de Moura, Mirceli Goulart Barbosa, Daiana Barbosa da Silva, Tarissa Ribeiro, Cláudia Eugênio, Daniel Schneider, Débora Mariani, Rodrigo Wiltgen Jeffman, Fernando Bozza, Alexandre Biasi Cavalcanti, Luciano Cesar Pontes Azevedo, Flávia Ribeiro Machado, Jorge Ibrain Salluh, José Augusto Santos Pellegrini, Rafael Barberena Moraes, Lucas Petri Damiani, Nilton Brandão da Silva, Maicon Falavigna, Regis Goulart Rosa

**Affiliations:** 1 Research Projects Office, Hospital Moinhos de Vento (HMV), Rua Ramiro Barcelos, 910, Moinhos de Vento, Porto Alegre, RS 90035-001 Brazil; 2 Intensive Care Unit, HMV. Rua Ramiro Barcelos, 910, Moinhos de Vento, Porto Alegre, RS 90035-001 Brazil; 3 Department of Critical Care, Instituto D’Or de Pesquisa e Ensino (IDOR), Rua Diniz Cordeiro, 30, Botafogo, Rio de Janeiro, RJ 22281-100 Brazil; 4 HCor Research Institute, Rua Abílio Soares, 250, Paraíso, São Paulo, SP 04005-909 Brazil; 5 Intensive Care Unit, Hospital Sírio-Libanês, Rua Dona Adma Jafet, 91, Bela Vista, São Paulo, SP 01308-050 Brazil; 6 Department of Anesthesiology, Pain and Intensive Care, Universidade Federal de São Paulo (UNIFESP), Rua Napoleão de Barros 737, Vila Clementino, São Paulo, SP 04024-900 Brazil; 7 Intensive Care Unit, Hospital de Clínicas de Porto Alegre (HCPA), Rua Ramiro Barcelos, 2350, Santa Cecília, Porto Alegre, RS 90035-903 Brazil; 8 Department of Internal Medicine, School of Medicine, Universidade Federal de Ciências da Saúde de Porto Alegre (UFCSPA), Rua Sarmento Leite, 245, Centro Histórico, Porto Alegre, RS 90050-170 Brazil; 9 Institute for Education and Research, HMV, Rua Ramiro Barcelos, 910, Moinhos de Vento, Porto Alegre, RS 90035-001 Brazil

**Keywords:** Critical care, Delirium, Cross infection, Family, Personal satisfaction, Anxiety, Depression, Health personnel, Burnout

## Abstract

**Background:**

Most adult intensive care units (ICUs) worldwide adopt restrictive family visitation models (RFVMs). However, evidence, mostly from non-randomized studies, suggests that flexible adult ICU visiting hours are safe policies that can result in benefits such as prevention of delirium and increase in satisfaction with care. Accordingly, the ICU Visits Study was designed to compare the effectiveness and safety of a flexible family visitation model (FFVM) vs. an RFVM on delirium prevention among ICU patients, and also to analyze its potential effects on family members and ICU professionals.

**Methods/design:**

The ICU Visits Study is a cluster-randomized crossover trial which compares an FFVM (12 consecutive ICU visiting hours per day) with an RFVM (< 4.5 ICU visiting hours per day) in 40 Brazilian adult ICUs. Participant ICUs are randomly assigned to either an FFVM or RFVM in a 1:1 ratio. After enrollment and follow-up of 25 patients, each ICU is crossed over to the other visitation model, until 25 more patients per site are enrolled and followed. The primary outcome is the cumulative incidence of delirium measured by the Confusion Assessment Method for the ICU. Secondary and tertiary outcomes include relevant measures of effectiveness and safety of ICU visiting policies among patients, family members, and ICU professionals. Herein, we describe all primary statistical procedures that will be used to evaluate the results and perform exploratory and sensitivity analyses of this study. This pre-specified statistical analysis plan was written and submitted without knowledge of the study data.

**Discussion:**

This a priori statistical analysis plan aims to enhance the transparency of our study, facilitating unbiased analyses of ICU visit study data, and provide guidance for statistical analysis for groups conducting studies in the same field.

**Trial registration:**

ClinicalTrials.gov, NCT02932358. Registered on 11 October 2016.

## Background

The recognition of the important role of family members in the intensive care unit (ICU) is the cornerstone of patient-centered care [1]. Beyond the justification of humanization, this strategy is proposed as a means to improve patient and family member outcomes [2]. In this context, small studies with a predominant before and after design have shown an association between flexible ICU visiting hours and reduced incidence of delirium in patients and increased satisfaction with care in family members [3–5]. Despite the growing recognition of the importance of family presence in the ICU, most ICUs around the world still adopt restricted visiting hours motivated by the ICU professionals’ perceptions of increased risk of disorganization of care, infection transmission, and burnout [6–9]. Unfortunately, few randomized studies have focused on the evaluation of the impact of different ICU visiting models on patients, family members, and ICU professionals, and this lack of evidence may constitute a barrier to the implementation of patient-centered care interventions in the ICU. The ICU Visits Study aims to investigate the effectiveness and safety of a flexible family visitation model (FFVM) vs. a restrictive family visitation model (RFVM) on delirium prevention among ICU patients, and to evaluate its potential benefits and hazards for family members and ICU professionals.

The present statistical analysis plan (SAP) aims to describe the trial’s analytical objectives and procedures before the end of the study recruitment and locking of the trial database to start analyses to comply with good clinical practice and avoid outcome reporting bias. This SAP was drafted without knowledge of any of the results of the investigators.

## Trial overview

**Fig. 1:**
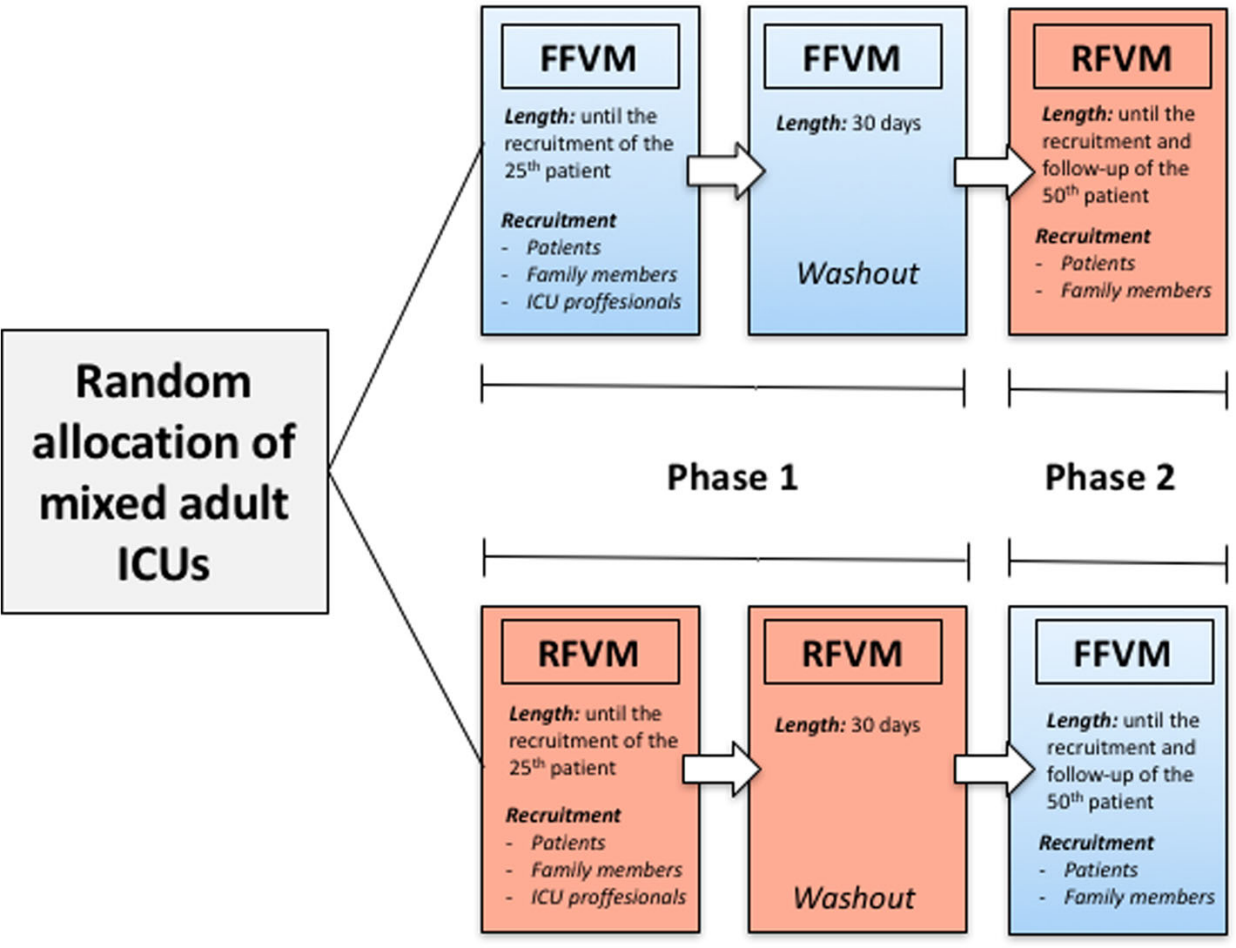
Study design. *FFVM* flexible family visitation model, *ICU* intensive care unit, *RFVM* restrictive family visitation model. All ICUs will have a learning period within the first 15 days of phases 1 and 2. During this period, ICUs will receive the intervention (FFVM or RFVM) but will not recruit subjects. Local investigators will use this period to adapt the ICU staff to the organizational aspects of study interventions

The ICU Visits Study is a cluster-randomized, crossover trial comparing an FFVM (12 consecutive ICU visiting hours per day) with an RFVM (< 4.5 ICU visiting hours per day) in Brazilian adult ICUs. The study background, design, rationale, eligibility criteria, and sample size have been previously published [10]. In brief, mixed adult ICUs of public and private philanthropic hospitals with a restrictive policy of ICU visiting hours (< 4.5 h/day) are randomly assigned to either an FFVM or RFVM in a 1:1 ratio until the recruitment and follow-up of 25 patients (phase 1) (Fig. 1). After a 30-day washout period without subject recruitment, each ICU is switched over to the other visitation model (phase 2) until 25 more patients per site are enrolled and followed. The randomization is stratified by the number of ICU beds (1 to 10 or > 10 ICU beds) and performed using random block sizes of 2, 4, and 6. Consecutive patients aged ≥18 years admitted to the ICU, their closest family members, and bedside ICU professionals in each cluster are considered eligible for the present trial. A complete description of the inclusion and exclusion criteria is shown in Table 1.

**Table 1:** Eligibility criteria

	Inclusion criteria	Exclusion criteria
Cluster	Adult medical-surgical ICUs of public and philanthropic hospitals with at least six beds; restrictive policy of ICU visiting hours (< 4.5 h/day)	Structural or organizational impediments to flexible family visitation, according to the Brazilian resolution of minimal operational requirements for ICUs^a^
Patients	ICU patients aged ≥18 years	Coma (RASS − 4 or − 5) lasting > 96 h from the moment of first evaluation for recruitment; delirium at baseline (positive CAM-ICU); cerebral death; aphasia; severe hearing deficit; predicted ICU length of stay < 48 h; exclusive palliative treatment at ICU admission; unavailability of a family member to participate in the flexible family visits; unlikelihood to survive > 24 h; prisoner status; readmission to the ICU after enrollment in the study
Family members	Closest family member of a patient enrolled in the study	Family members who do not speak Portuguese or have serious difficulty in answering the self-applied questionnaires (e.g., due to illiteracy or severe visual or hearing limitations); having another family member already enrolled in the study
ICU professionals	Bedside ICU professionals (physicians, nurses, nursing technicians, and physiotherapists) who assist patients during daytime for at least 20 h/week at the enrolled ICU	ICU professionals who have a planned leave of absence of > 15 days during phase 1 of the study

*CAM-ICU* Confusion Assessment Method for the Intensive Care Unit, *ICU* intensive care unit, *RASS* Richmond Agitation-Sedation Scale

^a^Available from: http://bvsms.saude.gov.br/bvs/saudelegis/anvisa/2010/res0007_24_02_2010.html. Accessed 26 Aug 2017

In the FFVM, patients are allowed to receive visits from one or two close family members for up to 12 consecutive hours each day. Family members who agree to join the family visits have to attend a structured meeting at the ICU in which they receive guidance about the ICU environment, common ICU treatments, rehabilitation and basic infection control practices, multidisciplinary work at the ICU, and information on palliative care and delirium prevention. Additionally, family members receive an information brochure and are encouraged to access a website, both of which are designed to explain what happens during and after an ICU stay to legitimize emotions and improve cooperation with relatives without increasing the ICU staff workload. In addition to family visitation, patients in the FFVM are allowed to receive social visits at specific time intervals (according to the local ICU policies) from friends or other family members who did not qualify for flexible family visitation. In the RFVM, patients are allowed visitors according to routine ICU practices, but limited to the maximum of 4.5 h of visitation per day. Visitors are not required to attend the structured meeting in the RFVM.

The study primary outcome is the cumulative incidence of delirium during the ICU stay measured by trained researchers using the Confusion Assessment Method for the ICU (CAM-ICU) [11]. Secondary outcomes include daily hazard of delirium, ventilator-free days at day 7, any ICU-acquired infections according to the Centers for Disease Control and Prevention (CDC) criteria [12–14], ICU length of stay, and hospital mortality among the patients; symptoms of anxiety and depression measured by the Hospital Anxiety and Depression Scale (HADS) [15] and satisfaction measured by the Critical Care Family Needs Inventory (CCFNI) [16] among the family members; and prevalence of burnout syndrome evaluated by the Maslach Burnout Inventory (MBI) [17] among the ICU professionals. Tertiary outcomes include need for antipsychotic agents, need for mechanical restraints, unplanned loss of invasive devices (venous catheter, enteral tube, or urinary catheter), coma-free days at day 7, and ICU-acquired pneumonia, urinary tract infection, or bloodstream infection among the patients; self-perception of involvement in patient care among the family members; and satisfaction among the ICU professionals.

## Statistical analysis plan

### Overall principles

The main analysis for each outcome will be performed at the subject level using the intention-to-treat (ITT) principle, meaning that all participants with a recorded outcome will be included in the analysis and will be analyzed according to the treatment group to which they were randomized (FFVM or RFVM), independent of actual visiting hours. Moreover, all analyses will account for the cluster-randomized crossover design to ensure correct type I error rates and confidence intervals (CIs). A significance level of 0.05, adjusted for multiple comparisons when appropriate, will be used for all statistical comparisons. Analysis will start once all data to discharge for the last included patient have been obtained, the database has been cleaned and locked, and the SAP has been submitted for publication. The R Development Core Team software will be used for analysis [18].

### Handling of missing data

We anticipate minimal missing values, given that the study variables will be determined during hospital stay by trained researchers. Nevertheless, the coordinator center will contact site investigators to retrieve any missing data values.

ICUs with incomplete subject recruitment (e.g., less than 50 patients) will be included in the primary analysis for the study outcomes considering all subjects available in the cluster. To assess the risk of bias related to ICUs that did not achieve the patient recruitment goal, multiple imputation techniques will be performed for the primary outcome and presented as sensitivity analyses. The missing values for the variables that compose the HADS, CCFNI, and MBI will be imputed, replacing the missing items with the mean of the answered items in the same subscale, if at least half of that subscale has been answered. The missing values for the variables that compose the PREdiction of DELIRium in ICU patients (PRE-DELIRIC) score will be imputed in a similar way as in the original study [19]. We will assume that if a variable is not determined, most likely the missing variable has a normal or negative value (e.g., no infection, no metabolic acidosis) or a mean value (e.g., Acute Physiology and Chronic Health Evaluation-II [APACHE-II] score) of the study population. We will not perform any imputation for the length of ICU visits per patient per day.

### Definition of analysis sets

At the cluster level, the ITT population includes all randomized ICUs that recruited subjects, regardless of the degree of adherence to the study interventions or achievement of the patient recruitment goal (50 patients per ICU). At the subject level, the ITT population includes all participants, regardless of protocol deviations. This includes patients who did not receive any ICU visits and family members who did not visit patients during the ICU stay, as well as patients who received visits longer than the maximum limit of visiting hours in both study periods (FFVM and RFVM).

### Statistical analyses

#### Patient flow

The flow of participants will be displayed in accordance with the Consolidated Standards of Reporting Trials (CONSORT) flow diagram (CONSORT 2010 statement: extension to cluster-randomized trials) [20]. This description will include information about eligibility criteria and follow-up losses at both cluster and subject levels.

#### Adherence to study interventions

The total length of visits per patient per day will be evaluated as the primary adherence variable to the study interventions. We will consider the length of stay of all visitors (whether family members or not) at the bedside of an enrolled patient during the ICU stay. For analytical purposes, we will not consider overlapping visiting hours in the total length of visits per patient per day (i.e., only the period in which there was at least one visitor [apart from the number of visitors] will be considered). The differences in the means of length of visits per patient per day between the FFVM and the RFVM groups will be compared using generalized estimating equations with adjustment for the cluster effect, period effect, and interaction between cluster and period. Additionally, we will describe the FFVM family members’ adherence to the website, and the healthcare professionals’ perception of effectiveness and barriers for implementation of the FFVM.

To assess the fidelity of FFVM implementation, we will perform on-site monitoring visits in all participant ICUs. During these visits, the ICU staff perception about adherence to proposed FFVM processes will be assessed by the coordinating center researchers using semi-structured interviews. During the FFVM period, each ICU will be rated from 0 to 100% (with higher percentages indicating higher levels of adherence to intended FFVM processes) in the following domains: (1) Visiting hours - adherence to visiting hours according to the study protocol; (2) Dissemination - dissemination of the FFVM to family members of ICU patients; (3) Structured meetings - frequency and quality of structured meetings; (4) Staff training - education of the ICU staff about the FFVM procedures. The total fidelity of the FFVM implementation score represents the mean of the four evaluated domains. We plan to perform sensitivity and exploratory analyses considering the effects of FFVM implementation on outcomes.

#### Baseline characteristics

The baseline characteristics of all participants will be presented by study arm in a table (see Table 2), but no formal statistical hypothesis testing will be performed to avoid unnecessary testing.

**Table 2:** Baseline characteristics of study participants

	FFVM	RFVM
Patients		
Age, years: mean (SD)	xx.x (xx.x)	xx.x (xx.x)
Age ≥ 65 years: *n*/total *n* (%)	x/x (xx.x)	x/x (xx.x)
Female gender: *n*/total *n* (%)	x/x (xx.x)	x/x (xx.x)
Charlson comorbidity index: median (IQR)	xx.x (xx.x-xx.x)	xx.x (xx.x-xx.x)
History of dementia: *n*/total *n* (%)	x/x (xx.x)	x/x (xx.x)
Hazardous alcohol consumption:^a^ *n*/total *n* (%)	x/x (xx.x)	x/x (xx.x)
ICU admission type		
Medical: *n*/total *n* (%)	x/x (xx.x)	x/x (xx.x)
Emergency surgery: *n*/total *n* (%)	x/x (xx.x)	x/x (xx.x)
Elective surgery: *n*/total *n* (%)	x/x (xx.x)	x/x (xx.x)
PRE-DELIRIC score: mean (SD)	xx.x (xx.x)	xx.x (xx.x)
APACHE-II score:^b^ mean (SD)	xx.x (xx.x)	xx.x (xx.x)
SOFA score:^b^ mean (SD)	xx.x (xx.x)	xx.x (xx.x)
Mechanically ventilated: *n*/total *n* (%)	x/x (xx.x)	x/x (xx.x)
Use of vasopressor: *n*/total *n* (%)	x/x (xx.x)	x/x (xx.x)
Use of corticosteroids: *n*/total *n* (%)	x/x (xx.x)	x/x (xx.x)
Use of parenteral sedative:^b^ *n*/total *n* (%)	x/x (xx.x)	x/x (xx.x)
Use of benzodiazepine:^b^ *n*/total *n* (%)	x/x (xx.x)	x/x (xx.x)
Use of opioid:^b^ *n*/total *n* (%)	x/x (xx.x)	x/x (xx.x)
Indwelling central venous catheter: *n*/total *n* (%)	x/x (xx.x)	x/x (xx.x)
Urinary catheter: *n*/total *n* (%)	x/x (xx.x)	x/x (xx.x)
Family members		
Age, years: mean (SD)	xx.x (xx.x)	xx.x (xx.x)
Female gender: *n*/total *n* (%)	x/x (xx.x)	x/x (xx.x)
Years of education: mean (SD)	xx.x (xx.x)	xx.x (xx.x)
Household income, USD: median (IQR)	xx.x (xx.x-xx.x)	xx.x (xx.x-xx.x)
Unemployed: *n*/total *n* (%)	x/x (xx.x)	x/x (xx.x)
Living with care recipient: *n*/total *n* (%)	x/x (xx.x)	x/x (xx.x)
Surrogate decision maker: *n*/total *n* (%)	x/x (xx.x)	x/x(xx.x)
History of anxiety: *n*/total *n* (%)	x/x (xx.x)	x/x (xx.x)
History of depression: *n*/total *n* (%)	x/x (xx.x)	x/x (xx.x)
ICU professionals		
Age, years: mean (SD)	xx.x (xx.x)	xx.x (xx.x)
Female gender: *n*/total *n* (%)	x/x (xx.x)	x/x (xx.x)
Type of ICU professional		
Physician: *n*/total *n* (%)	x/x (xx.x)	x/x (xx.x)
Nurse: *n*/total *n* (%)	x/x (xx.x)	x/x (xx.x)
Nurse technician: *n*/total *n* (%)	x/x (xx.x)	x/x (xx.x)
Physiotherapist: *n*/total *n* (%)	x/x (xx.x)	x/x (xx.x)
Years of experience in ICU: mean (SD)	xx.x (xx.x)	xx.x (xx.x)
Hours of work per week: mean (SD)	xx.x (xx.x)	xx.x (xx.x)
Number of patients per professional		
Physician: mean (SD)	xx.x (xx.x)	xx.x (xx.x)
Nurse: mean (SD)	xx.x (xx.x)	xx.x (xx.x)
Nurse technician: mean (SD)	xx.x (xx.x)	xx.x (xx.x)
Physiotherapist: mean (SD)	xx.x (xx.x)	xx.x (xx.x)
Burnout syndrome at baseline:^c^ *n*/total *n* (%)	x/x (xx.x)	x/x (xx.x)

*APACHE-II* Acute Physiology and Chronic Health Evaluation-II, *FFVM* flexible family visitation model, *ICU* intensive care unit, *IQR* interquartile range (P25–P75), *PRE-DELIRIC* PREdiction of DELIRium in ICU patients, *RFVM* restrictive family visitation model, *SD* standard deviation, *SOFA* Sequential Organ Failure Assessment, *USD* United States dollars

^a^Alcohol consumption greater than or equal to 14 units per week for women and greater than or equal to 21 units per week for men

^b^Within the first 24 h of inclusion in the study

^c^Maslach Burnout Inventory total score > − 9

#### Inter-rater reliability of the CAM-ICU

Inter-rater reliability measures of the CAM-ICU will be performed before study initiation to evaluate the quality of assessment. In each participant ICU, we will assess the concordance and agreement in diagnostic classification between trained intensive care physicians (reference diagnosis) and local outcome evaluators.

#### Primary outcome

All the pre-specified study outcomes are described in Table 3. The primary outcome is the cumulative incidence of delirium during ICU stay determined by the CAM-ICU, which was validated for the Brazilian population of critical care patients [21]. The cumulative incidence of delirium is defined as the presence of delirium (at least one positive CAM-ICU score) during the ICU stay. The differences in the incidences of delirium between the FFVM and the RFVM groups will be compared using generalized estimating equations with adjustment for the cluster effect, period effect, and interaction between intervention and period and presented as risk ratio (RR) and 95% CI.

**Table 3:** Study outcomes

Outcomes	FFVM	RFVM	Type of effect estimate	Effect estimate (CI)	*p* value^a^
Primary					
Cumulative incidence of delirium:^b^ *n*/total *n* (%)	x/x (xx.x)	x/x (xx.x)	RR	x.xx (x.xx-x.xx)*	x.xx
Secondary					
Patients					
Daily hazard of delirium:^b^ mean (SD)	xx.x (xx.x)	xx.x (xx.x)	HR	x.xx (x.xx-x.xx)**	x.xx
Any ICU-acquired infection:^c^ *n*/total *n* (%)	x/x (xx.x)	x/x (xx.x)	RR	x.xx (x.xx-x.xx)*	x.xx
Proportion of ventilator free-days: mean (SD)	xx.x (xx.x)	xx.x (xx.x)	MD	x.xx (x.xx-x.xx)**	x.xx
ICU length of stay: mean (SD)	xx.x (xx.x)	xx.x (xx.x)	MD	x.xx (x.xx-x.xx)**	x.xx
Hospital mortality: *n*/total *n* (%)	x/x (xx.x)	x/x (xx.x)	RR	x.xx (x.xx-x.xx)**	x.xx
Family members					
HADS anxiety score: mean (SD)	xx.x (xx.x)	xx.x (xx.x)	MD	x.xx (x.xx-x.xx)***	x.xx
HADS depression score: mean (SD)	xx.x (xx.x)	xx.x (xx.x)	MD	x.xx (x.xx-x.xx)***	x.xx
CCFNI satisfaction score: mean (SD)	xx.x (xx.x)	xx.x (xx.x)	MD	x.xx (x.xx-x.xx)***	x.xx
ICU professionals					
Burnout syndrome:^d^ *n*/total *n* (%)	x/x (xx.x)	x/x (xx.x)	PR	x.xx (x.xx-x.xx)*	x.xx
Tertiary					
Patients					
Need for antipsychotic agents: *n*/total *n* (%)	x/x (xx.x)	x/x (xx.x)	RR	x.xx (x.xx-x.xx)*	x.xx
Need for mechanical restraints: *n*/total *n* (%)	x/x (xx.x)	x/x (xx.x)	RR	x.xx (x.xx-x.xx)*	x.xx
Unplanned loss of invasive devices: *n*/total *n* (%)	x/x (xx.x)	x/x (xx.x)	RR	x.xx (x.xx-x.xx)*	x.xx
Proportion of coma-free days: mean (SD)	xx.x (xx.x)	xx. (xx.x)	MD	x.xx (x.xx-x.xx)*	x.xx
ICU-acquired pneumonia:^c^ *n*/total *n* (%)	x/x (xx.x)	x/x (xx.x)	RR	x.xx (x.xx-x.xx)*	x.xx
ICU-acquired UTI:^c^ *n*/total *n* (%)	x/x (xx.x)	x/x (xx.x)	RR	x.xx (x.xx-x.xx)*	x.xx
ICU-acquired BSI:^c^ *n*/total *n* (%)	x/x (xx.x)	x/x (xx.x)	RR	x.xx (x.xx-x.xx)*	x.xx
Family members					
Self-perception of involvement in patient care					
Score:^e^ mean (SD)	xx.x (xx.x)	xx.x (xx.x)	MD	x.xx (x.xx-x.xx)*	x.xx
ICU professionals					
Satisfaction of ICU professionals with the ICU visiting policy score:^f^ mean (SD)	xx.x (xx.x)	xx.x (xx.x)	MD	x.xx (x.xx-x.xx)*	x.xx

*BSI* bloodstream infection, *CCFNI* Critical Care Family Needs Inventory, *CI* confidence interval, *FFVM* flexible family visitation model, *HADS* Hospital Anxiety and Depression Scale, *HR* hazard ratio, *ICU* intensive care unit, *MD* mean difference, *PR* prevalence ratio, *RFVM* restrictive family visitation model, *RR* risk ratio, *SD* standard deviation, *UTI* urinary tract infection

^a^Adjusted for multiple comparisons with Bonferroni correction when appropriate

^b^According to the Confusion Assessment Method for the Intensive Care Unit (CAM-ICU) criteria

^c^According to the Centers for Disease Control and Prevention (CDC) criteria

^d^Maslach Burnout Inventory (MBI) total score > − 9

^e^The self-perception of involvement in patient care score varies from 0 (no involvement) to 27 (maximum degree of involvement)

^f^The satisfaction of ICU professionals with the ICU visiting policy score varies from 0 (unsatisfied) to 4 (very satisfied)

^*^95% confidence interval

^**^99% confidence interval

^***^98.3% confidence interval

#### Sensitivity analyses for the primary outcome

We aim to conduct the following sensitivity analyses for the primary outcome to assess consistency and the risk of bias:

1. Evaluation of study intervention effects adjusted by the baseline risk of delirium according to the PRE-DELIRIC score.
2. Evaluation of study intervention effects considering the cluster adherence to FFVM implementation. In this analysis, the study intervention effects will be adjusted by the total fidelity of FFVM implementation score.
3. Evaluation of study intervention effects considering the potential confounding effect of sedation on the delirium diagnosis. In this analysis, the study intervention effects will be evaluated, considering patients with positive CAM-ICU during ICU stay in the context of Richmond Agitation-Sedation Scale (RASS) score – 2 or − 3 as non-delirious subjects.
4. Evaluation of effects of study interventions, considering a best plausible scenario/worst plausible scenario imputation of outcomes among ICUs that did not complete the patient recruitment as follows:
  - *Best plausible scenario*: Imputation of the lowest study cluster/phase incidence of delirium for missing FFVM patients and the highest study cluster/phase incidence of delirium for missing RFVM patients
  - *Worst plausible scenario*: Imputation of the lowest study cluster/phase incidence of delirium for missing RFVM patients and the highest study cluster/phase incidence of delirium for missing FFVM patients.

#### Subgroup analyses for the primary outcome

There will be three a priori defined subgroup analyses for the primary endpoint: (1) effectiveness of the FFVM vs. RFVM in ICUs according to the PRE-DELIRIC score (patients with a predicted risk < 25%, 25–50%, 50–75%, and > 75%); (2) effectiveness of the FFVM vs. RFVM according to patient group (medical vs. surgical and neurocritical vs. non-neurocritical); and (3) effectiveness of the FFVM vs. RFVM in ICUs according to APACHE-II scores (≤15 vs. > 15 points). The consistency of intervention effects across the above-mentioned subgroups will be assessed by means of tests for interaction. The Bonferroni correction will be applied to adjust the subgroup analyses for multiple comparisons.

#### Secondary outcomes

We will use the following statistical procedures to evaluate the study secondary outcomes:

1. *Daily hazard of delirium*: The daily hazard of delirium will be analyzed using a joint survival model that accounts for the treatment effect (with adjustment for the cluster effect, period effect, and interaction between cluster and period) on repeated daily indicator of delirium within each patient and terminating event (death or discharge from the ICU) [22]. This outcome will be presented as hazard ratio (HR) and 95% CI.
2. *Any ICU-acquired infections*: The differences in the cumulative incidence of any ICU-acquired infection between the FFVM and RFVM groups will be compared using generalized estimating equations with adjustment for the cluster effect, period effect, and interaction between intervention and period. This outcome will be presented as RR and 95% CI.
3. *Ventilator-free days*: The number of ventilator-free days will be evaluated using the 7-day time horizon. Ventilator-free days will be set to 0 for patients who have died. The differences in the mean ventilator-free days between the FFVM and RFVM groups will be compared using generalized estimating equations with adjustment for the cluster effect, period effect, and interaction between intervention and period. This outcome will be presented as mean difference (MD) and 95% CI.
4. *ICU length of stay*: The differences in the mean ICU length of stay between the FFVM and RFVM groups will be compared using generalized estimating equations with adjustment for the cluster effect, period effect, and interaction between intervention and period. This outcome will be presented as MD and 95% CI.
5. *Hospital mortality*: The differences in hospital mortality rates between the FFVM and RFVM groups will be compared using generalized estimating equations with adjustment for the cluster effect, period effect, and interaction between intervention and period. This outcome will be presented as RR and 95% CI.
6. *Symptoms of anxiety and depression among family members*: The differences in the mean HADS scores for anxiety and depression between the FFVM and RFVM groups will be compared using generalized estimating equations with adjustment for the cluster effect, period effect, and interaction between intervention and period. These outcomes will be presented as MD and 95% CI.
7. *Satisfaction among family members*: The differences in the mean CCFNI satisfaction scores between the FFVM and RFVM groups will be compared using generalized estimating equations with adjustment for the cluster effect, period effect, and interaction between intervention and period. This outcome will be presented as MD and 95% CI.
8. *Prevalence of burnout among the ICU professionals*: Burnout is defined as a cumulative MBI score greater than − 9. The differences in the prevalence of burnout between the FFVM and RFVM groups will be compared using generalized estimating equations with adjustment for the cluster effect and baseline MBI total scores. This outcome will be presented as prevalence ratio and 95% CI.

Given that the main safety outcomes of the present study are any ICU-acquired infections and burnout, we will not adjust these outcomes for multiple comparisons. For other secondary outcomes, the Bonferroni correction will be applied to adjust the analyses for multiple comparisons when appropriate, taking into consideration the number of comparisons within each population of interest (patients, family members, and ICU professionals).

We will perform sensitivity analyses to check the consistency of intervention effects on family members and ICU professionals as follows:

1. Evaluation of study intervention effects on HADS subscale scores among family members, considering HADS scores as categorical variables with established cutoff points (> 10 points for both anxiety and depression subscales)
2. Evaluation of study intervention effects on HADS subscale scores among family members adjusted by previous history of anxiety (for anxiety HADS subscale) and depression (for depression HADS subscale)
3. Evaluation of study intervention effects on CCFNI subscale domains (information, proximity, reassurance, support, and comfort)
4. Evaluation of study intervention effects on incidence (excluding individuals with burnout at baseline) and prevalence of burnout among ICU professionals, considering the following alternative MBI criteria:
  - *Alternative criteria 1*: An emotional exhaustion subscale score ≥ 27, OR a depersonalization subscale score ≥ 10, OR a personal accomplishment subscale score ≤ 33
  - *Alternative criteria 2*: An emotional exhaustion subscale score ≥ 27, OR a depersonalization subscale score ≥ 10
  - *Alternative criteria 3*: An emotional exhaustion subscale score ≥ 27, AND a depersonalization subscale score ≥ 10, AND a personal accomplishment subscale score ≤ 33.

#### Tertiary outcomes

We will use the following statistical procedures to evaluate the study tertiary outcomes:

1. *Need for antipsychotic agents*: The differences in the cumulative incidence of need for antipsychotic agents during ICU stay between the FFVM and RFVM groups will be compared using generalized estimating equations with adjustment for the cluster effect, period effect, and interaction between intervention and period. This outcome will be presented as RR and 95% CI.
2. *Need for mechanical restraints*: The differences in the cumulative incidence of need for mechanical restraints during ICU stay between the FFVM and RFVM groups will be compared using generalized estimating equations with adjustment for the cluster effect, period effect, and interaction between intervention and period. This outcome will be presented as RR and 95% CI.
3. *Unplanned loss of invasive devices*: The differences in the cumulative incidence of any unplanned loss of invasive devices (venous catheter, enteral tube, or urinary catheter) during the ICU stay between the FFVM and RFVM groups will be compared using generalized estimating equations with adjustment for the cluster effect, period effect, and interaction between intervention and period. This outcome will be presented as RR and 95% CI.
4. *Coma-free days at day 7*: The number of coma-free days will be evaluated using the 7-day time horizon. Coma-free days will be set to 0 for patients who have died. The differences in the mean proportion of days free of coma (RASS [23] – 4 or − 5) between the FFVM and RFVM groups will be compared using generalized estimating equations with adjustment for the cluster effect, period effect, and interaction between intervention and period. This outcome will be presented as MD and 95% CI.
5. *ICU-acquired pneumonia*: The differences in the cumulative incidence of ICU-acquired pneumonia between the FFVM and RFVM groups will be compared using generalized estimating equations with adjustment for the cluster effect, period effect, and interaction between intervention and period. This outcome will be presented as RR and 95% CI.
6. *ICU-acquired urinary tract infection*: The differences in the cumulative incidence of urinary tract infection between the FFVM and RFVM groups will be compared using generalized estimating equations with adjustment for the cluster effect, period effect, and interaction between intervention and period. This outcome will be presented as RR and 95% CI.
7. *ICU-acquired bloodstream infection*: The differences in the cumulative incidence of bloodstream infection between the FFVM and RFVM groups will be compared using generalized estimating equations with adjustment for the cluster effect, period effect, and interaction between intervention and period. This outcome will be presented as RR and 95% CI.
8. *Self-perception of involvement in patient care among the family members*: The self-perception of involvement in patient care will be evaluated using a score developed for the present study, which is composed of 9 questions related to the self-perception of involvement of the family member in the care of the patient in the following domains: (1) re-orientation activities, (2) pain control, (3) mobilization, (4) feeding, (5) hygiene, (6) emotional support, (7) helping patients to interpret ICU staff orientations, (8) helping the ICU professionals to understand patient needs, and (9) helping to create a patient-friendly environment in the patient room. The score of each question ranges from 0 (never involved in the activity) to 3 (very frequently involved in the activity). The total score, which varies from 0 (no involvement) to 27 (maximum degree of involvement), will be obtained by the sum of the scores of each question. The differences in the mean total scores of self-perception of involvement in patient care between the FFVM and RFVM groups will be compared using generalized estimating equations with adjustment for the cluster effect, period effect, and interaction between intervention and period. This outcome will be presented as MD and 95% CI.
9. *Satisfaction among ICU professionals*: The satisfaction of ICU professionals will be evaluated through the score of the following question: Are you satisfied with the current visiting policy of your ICU? The responses to this 5-option Likert scale question may be one of the following: 0 - unsatisfied, 1- somewhat dissatisfied, 2 - indifferent, 3 - somewhat satisfied, 4 - very satisfied. The differences in the mean score values between the FFVM and RFVM groups will be compared using generalized estimating equations with adjustment for the cluster effect. This outcome will be presented as MD and 95% CI.

No adjustment for multiple comparisons will be made for tertiary outcomes. Therefore, the results of tertiary outcomes should be considered exploratory.

### Differences between the study protocol and statistical analysis plan

There are no differences between the study protocol and this SAP in relation to the proposed outcomes or statistical procedures.

## Discussion and trial status

In this SAP, we present the statistical procedures that will allow the comparison of effectiveness and safety outcomes between the FFVM and RFVM in the ICU Visits Study. The present publication aims to avoid risks of outcome reporting bias and data-driven results and provide guidance for statistical analysis for future studies in this field. As of March 2018, 40 ICUs were randomized. Currently, 1591 patients, 1192 family members, and 829 ICU professionals were included in the study. We expect that the recruitment of subjects will be completed in June 2018.

## Abbreviations

APACHE-II: Acute Physiology and Chronic Health Evaluation-II
BSI: Bloodstream infection
CAM-ICU: Confusion Assessment Method for the Intensive Care Unit
CCFNI: Critical Care Family Needs Inventory
CDC: Centers for Disease Control and Prevention
CI: Confidence interval
CONSORT: Consolidated Standards of Reporting Trials
FFVM: Flexible family visitation model
HADS: Hospital Anxiety and Depression Scale
HR: Hazard ratio
ICU: Intensive care unit
IQR: Interquartile range
MBI: Maslach Burnout Inventory
MD: Mean difference
PR: Prevalence ratio
PRE-DELIRIC: PREdiction of DELIRium in ICU patients
RASS: Richmond Agitation-Sedation Scale
RFVM: Restrictive family visitation model
RR: Risk ratio
SAP: Statistical analysis plan
SD: Standard deviation
SOFA: Sequential Organ Failure Assessment
USD: United States dollars
UTI: Urinary tract infection
